# Complete Genome Sequence of *Campylobacter armoricus* CA639, Which Carries Two Plasmids, Compiled Using Oxford Nanopore and Illumina Sequencing Technologies

**DOI:** 10.1128/MRA.01309-19

**Published:** 2020-01-02

**Authors:** Amine M. Boukerb, Julien Schaeffer, Joëlle Serghine, Gregory Carrier, Françoise S. Le Guyader, Michèle Gourmelon

**Affiliations:** a IFREMER RBE-SGMM-LSEM, Laboratoire de Santé Environnement et Microbiologie, Plouzané, Institut Français de Recherche pour l’Exploitation de la Mer, France; b IFREMER RBE-BRM-LPBA, Laboratoire de Physiologie et Biotechnologie des Algues, Institut Français de Recherche pour l'Exploitation de la Mer, Nantes, France; DOE Joint Genome Institute

## Abstract

As determined by a hybrid approach combining Oxford Nanopore MinION and Illumina MiniSeq sequence data, *Campylobacter armoricus* strain CA639 harbored a circular chromosome of 1,688,169 bp with a G+C content of 28.47% and two plasmids named pCA639-1 and pCA639-2, with lengths of 51,123 and 28,139 bp, and G+C contents of 26.5% and 28.45%, respectively.

## ANNOUNCEMENT

Campylobacter armoricus is a novel urease-positive bacterial species phylogenetically classified within the Campylobacter lari group (1, 2). This group forms a distinct clade within the epsilon subdivision of the Proteobacteria and its members are among the thermotolerant Campylobacter spp. (3). We report here the complete sequence of the river water isolate *Campylobacter armoricus* CA639 and its native plasmids pCA639-1 and pCA639-2. This strain was isolated from the river Le Rat (La Fresnaye catchment, Brittany, France) on 4 March 2014 using the ISO-10272:2016 method (1, 2). Bacterial DNA was extracted from an overnight culture in trypto-casein-soy agar (bioMérieux, Marcy-l’Étoile, France) supplemented with 5% (vol/vol) sheep blood (Oxoid, Thermo Scientific, Inc.) at 42°C in a microaerobic atmosphere, using the DNA QIAamp minikit 250 (Qiagen, Venlo, The Netherlands) and used for Illumina and Nanopore sequencing. Genomic libraries were prepared using the Nextera DNA Flex library prep kit (Illumina, San Diego, CA, USA), and sequencing was performed on an Illumina MiniSeq platform with a 2 × 150 paired-end protocol (1). Default parameters were used for all software except where otherwise noted. Raw reads were quality filtered and adapter trimmed with Trimmomatic v.0.36 (4). An Oxford Nanopore Technologies (ONT) sequencing library was prepared using the manufacturer’s 1D genomic DNA by ligation kit (SQK-LSK 108), and sequencing was carried out on a MinION device using flow cell type R9.4.1 (FLO-MIN106D). Porechop v.0.2.1 (5) was used for adaptor trimming, and NanoFilt v.2.2.0 (6) was used to remove reads of <500 bp or with average quality scores of <10. Thus, we used a robust pipeline relying on a combination of Oxford Nanopore long-read (681,890; *N*_50_ value, 15,543 bp; 9.7 Gb of data) and Illumina short-read (1,309,028; 2 × 150-bp reads) technologies to scaffold and polish sequencing data.

**FIG 1:**
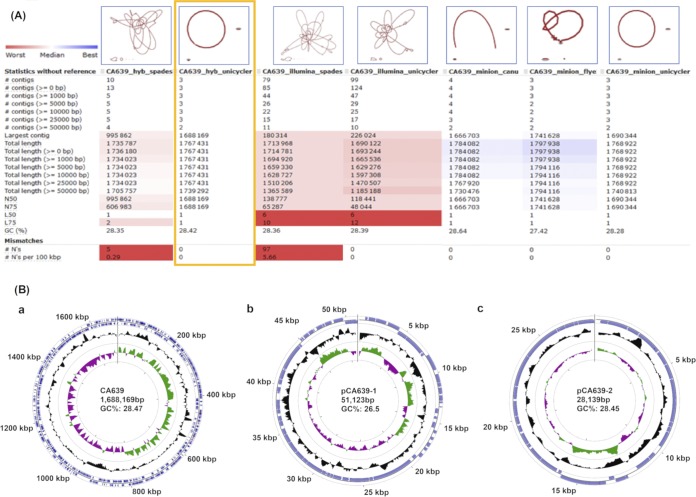
(A) Visualization of assembly graphs and statistics for each strategy was produced with Bandage v.0.8.1 (9) and QUAST v.5.0.0 (10), respectively. First, we constructed MiniSeq assemblies (illumina) using SPAdes v.3.12.0 (11) or Unicycler v.0.4.7 (12). Second, MinION assemblies (minion) were achieved using Canu v.1.5 (13), Flye v.2.4 (14), or Unicycler. These three assemblies were aligned to MinION reads using Minimap2 v.2.17 (15) and SAMtools v.1.9 (16) and then polished using Nanopolish v.0.11.0 (17). An additional round of Nanopolish did not improve their accuracy. Moreover, the Canu assembly was polished using Pilon v.1.23 (18) with the flags “–fix bases” and then “–fix all” by aligning MiniSeq reads using Bowtie 2 v.2.3.4.3 (19) and SAMtools. Third, we added MinION reads to the obtained MiniSeq-based assemblies to resolve ambiguous regions in the sequencing graph, creating SPAdes hybrid and Unicycler hybrid assemblies (Hyb). (B) Circular maps of the *C. armoricus* CA639 replicons (a, chromosome; b and c, plasmids) from the hybrid assembly using Unicycler were drawn using the online CGView server (http://stothard.afns.ualberta.ca/cgview_server/). Counting from the outside toward the center, circle 1 (outermost circle) shows distances from the putative origin of replication in kilobase pairs. Circle 2 shows annotated CDS (blue) encoded on the forward and reverse strands. The *rrs* operons and tRNA genes in the chromosome are indicated in pink and gray, respectively. Circle 3 shows G+C contents higher and lower than the average G+C content (black). Circle 4 shows G+C skew, with positive values in green and negative values in purple.

Several approaches were used to construct *de novo* assemblies using default parameters (Fig. 1). Based on the obtained statistics (Fig. 1A), the Unicycler hybrid assembly was selected for downstream analyses. This reported one circular chromosome of 1,688,169 bp (28.47% G+C content) and two plasmids named pCA639-1 and pCA639-2 with lengths of 51,123 and 28,139 bp and G+C contents of 26.5 and 28.45%, respectively (Fig. 1B). BBMap v.38.71 (https://sourceforge.net/projects/bbmap/) was used to calculate the average coverages for the chromosome (101.7× for short reads and 1,301.2× for long reads), pCA639-1 (87.9× and 328.9×, respectively), and pCA639-2 (178.8× and 280.4×, respectively).

Prokka v.1.14 (7) predicted 1,640 putative coding sequences (CDS), with 862 (52.6%) having assigned functions, including 3 rRNA operons and 43 tRNAs for the chromosome and 59 and 35 CDS for the two respective plasmids. The chromosome contains one prophage integrase and an ISHp*1* transposase (IS*1595* family). In addition to the results that were obtained for virulence (i.e., the *cdtABC* operon, *ciaB*, *flaC*, *porA*, and *cadF*) and antibiotic resistance (i.e., *cmeABC*, *cmeR*, *cosR*, *macAB, oxa-184*, and *oxa-493*) coding gene screening (1) using ABRicate v.0.8.7 (8), we detected a chloramphenicol acetyltransferase type III gene (*cat3*), a bicyclomycin resistance gene (*bcr*), and other multidrug efflux pump-coding genes that may be involved in antibiotic resistance. pCA639-1 harbored genes coding for the Tra/Vir type IV secretion system (T4SS) and a Cag pathogenicity island protein. A blastn search of the sequence of this plasmid against the NCBI database showed 79% query coverage and 94.56% identity with that of Campylobacter lari pCL2100 (GenBank accession number CP000933). pCA639-2 carried several conjugative transfer genes and shared 82% query coverage and 95.06% identity with pGMI16-001 (GenBank accession number CP028188) carried by Campylobacter coli strain CFSAN054106, suggesting an intraspecies dissemination.

This study highlights the value of combining short- and long-read sequencing data for high-quality genome assemblies and annotation of repetitive genomic regions. The complete genome sequence of C. armoricus CA639 comprises essential data for taxonomic and comparative genomic studies within a One Health approach, a concept which recognizes that the health of people is connected to the health of animals and the environment.

### Data availability.

The sequencing data have been deposited in the DDBJ/EMBL/GenBank databases under accession numbers CP044262 for the chromosome and CP044261 and CP044263 for plasmids pCA639-1 and pCA639-2, respectively. The Illumina paired-end fastq and ONT base-called fastq files are available in the Sequence Read Archive under accession numbers SRR10390899 and SRR10162491, respectively.
