# Correction for Boukerb et al., “Complete Genome Sequence of *Campylobacter armoricus* CA639, Which Carries Two Plasmids, Compiled Using Oxford Nanopore and Illumina Sequencing Technologies”

**DOI:** 10.1128/MRA.00045-20

**Published:** 2020-02-13

**Authors:** Amine M. Boukerb, Julien Schaeffer, Joëlle Serghine, Gregory Carrier, Françoise S. Le Guyader, Michèle Gourmelon

**Affiliations:** a IFREMER RBE-SGMM-LSEM, Laboratoire de Santé Environnement et Microbiologie, Plouzané, Institut Français de Recherche pour l’Exploitation de la Mer, France; b IFREMER RBE-BRM-LPBA, Laboratoire de Physiologie et Biotechnologie des Algues, Institut Français de Recherche pour l’Exploitation de la Mer, Nantes, France

## AUTHOR'S CORRECTION

Volume 9, no. 1, e01309-19, 2020, https://doi.org/10.1128/MRA.01309-19. Page 3: Line 3 of Acknowledgments should read as follows. “… and support services) and Catherine Ragimbeau from the Laboratoire National de Santé for providing Illumina raw reads.”

